# Supplementary material for: Mitochondrial Calcium Uptake Promotes Mitochondrial Activation and Hyperactivated Motility During Human Sperm Capacitation
Source: bioRxiv. 2026 Oct 2:2026.09.28.755036. Preprint. [Version 1] doi: 10.64898/2026.09.28.755036 (PMC13648570; doi:10.64898/2026.09.28.755036)
Supplement: Supplementary file 1 [file media-1.pdf]

Supplementary material.

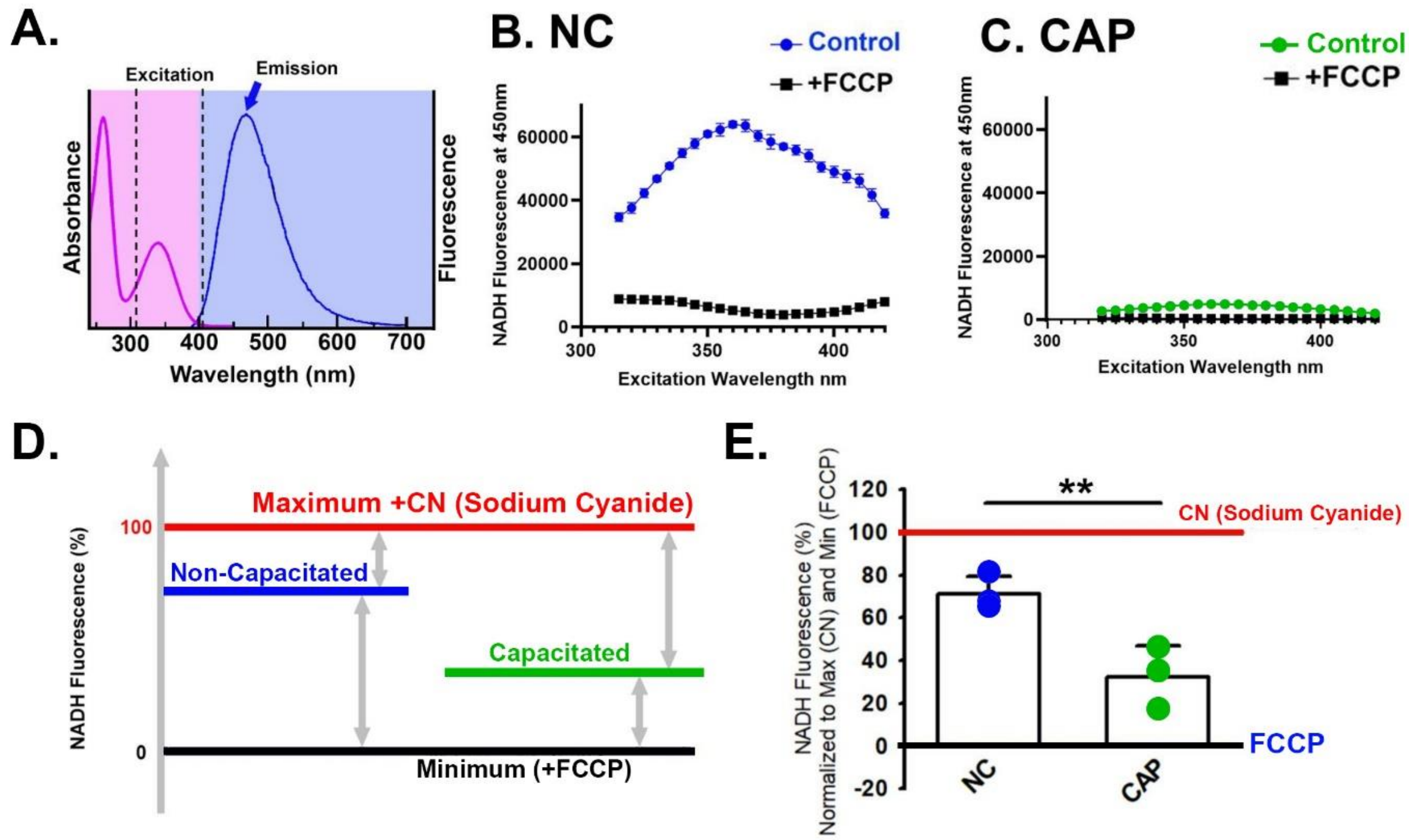

Supplementary Figure 1. Validation and quantification of NADH autofluorescence in non-capacitated and capacitated human spermatozoa. (**A**) Excitation and emission wavelengths used to measure NADH autofluorescence. (**B–C**) Excitation spectra of NADH autofluorescence recorded at an emission wavelength of 450 nm in non-capacitated (NC; blue, **B**) and capacitated (CAP; green, **C**) spermatozoa under control conditions and after FCCP treatment (black). These spectra were used to validate the excitation wavelength selected for subsequent NADH measurements. **(D)** Schematic representation of the fluorescence normalization approach using FCCP-induced minimum fluorescence and sodium cyanide (CN)-induced maximum fluorescence as reference points. **(E)** Quantification of NADH autofluorescence (λ_{ex} = 310 nm; λ_{em} = 450 nm) following normalization between the FCCP-defined minimum and CN-defined maximum. NC and CAP samples were obtained from the same donor and analyzed in parallel following the capacitation period. **P* < 0.05; ***P* < 0.01; ****P* < 0.001 by paired *t*-test.

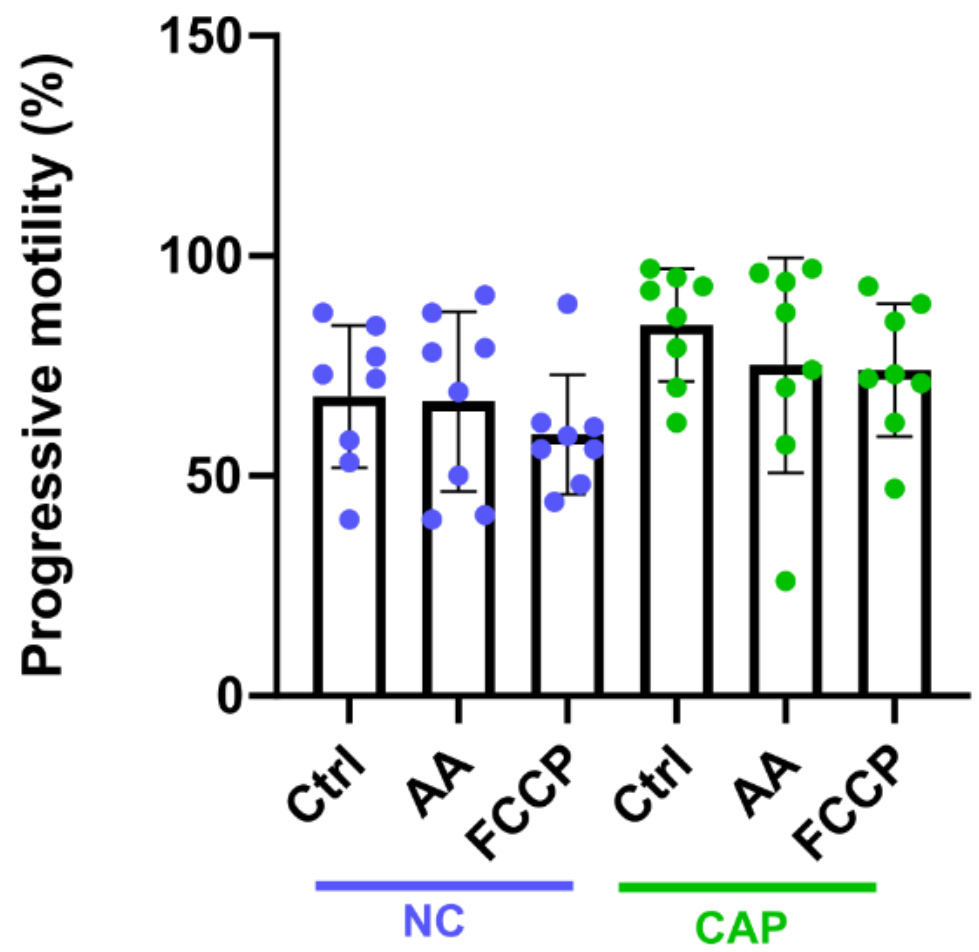

Supplementary Figure 2. Effects of mitochondrial disruption on sperm progressive motility (a+b,%). Spermatozoa motility was assessed by CASA at the Facultad de Medicina, Universidad de la República (UDELAR). **M**otility parameters were evaluated in the presence of the mitochondrial disruptors antimycin A (AA) and FCCP. *n* = 8. NC, non-capacitated (blue dots); CAP, capacitated (green dots). No significant differences were observed.

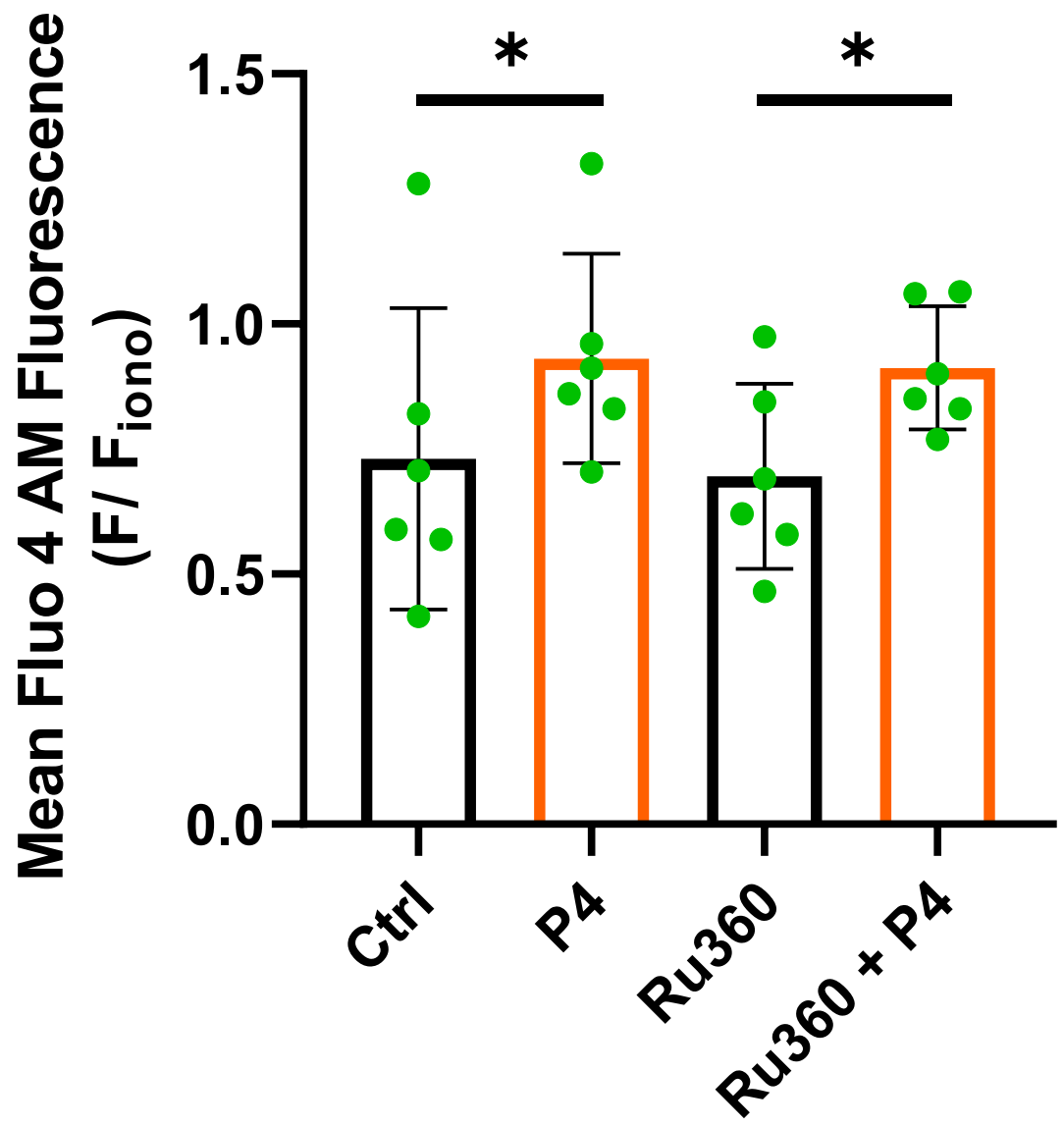

Supplementary Figure 3. Ru360 does not affect the progesterone-induced increase in intracellular Ca^{2+} in human spermatozoa. Intracellular Ca^{2+} was measured using Fluo-4 by flow cytometry in capacitated (CAP) spermatozoa in the absence (Ctrl) or presence of the MCU inhibitor Ru360 (n = 6) with or without Progesterone (P4; 30 μM). Data were acquired by flow cytometry at Washington University in St. Louis. *$P < 0.05$ by paired t -test.

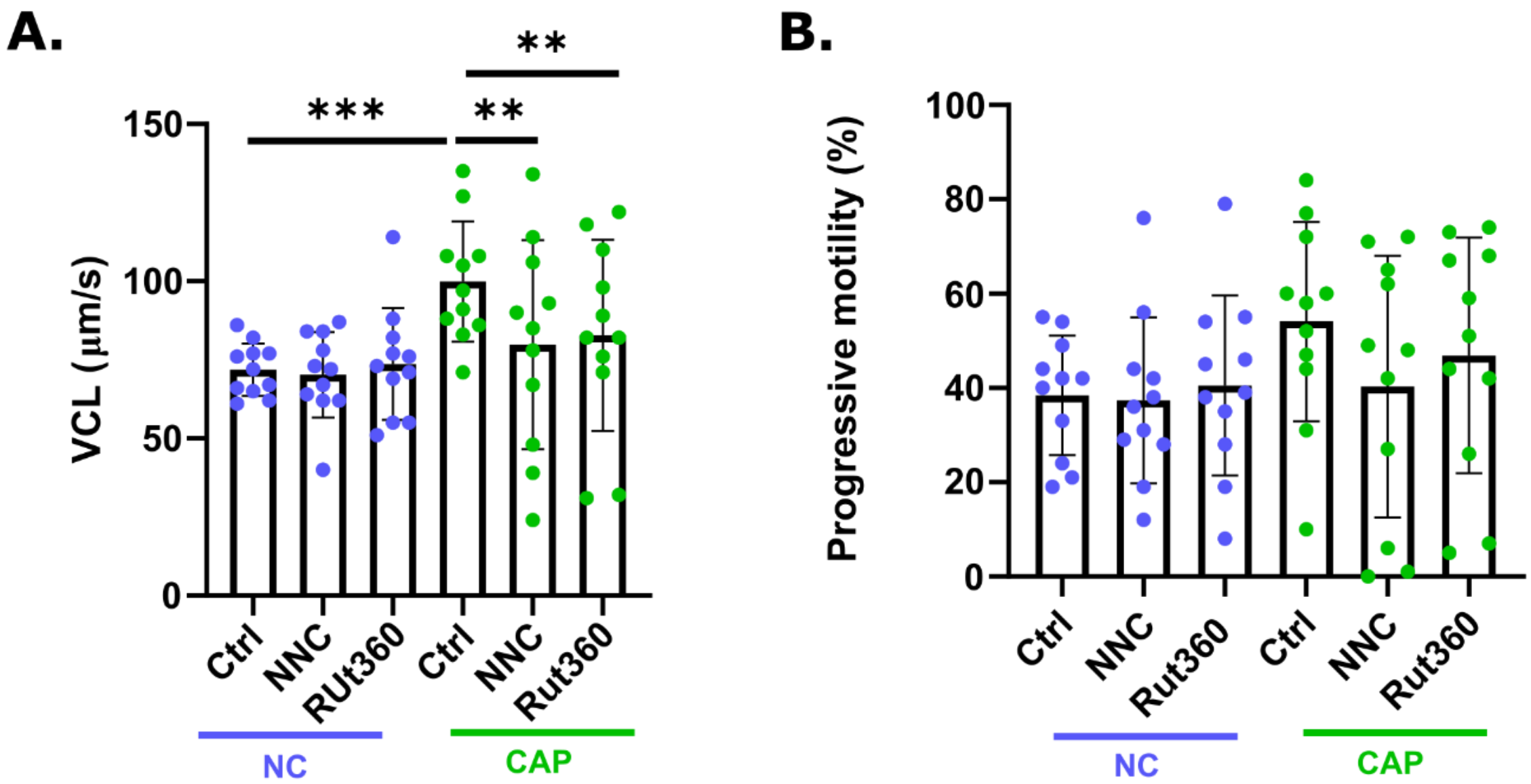

Supplementary Figure 4. Effects of Ca^{2+} channel inhibition on spermatozoa motility parameters. Spermatozoa motility was assessed by CASA at Washington University in St. Louis (WashU) Motility parameters were evaluated in the presence of the CatSper inhibitor NNC 55-0396 (NNC) and the MCU inhibitor Ru360. **(A)** Curvilinear velocity (VCL), n=11. **(B)** Progressive motility (a+b, %), *n* = 11. NC, non-capacitated (blue dots); CAP, capacitated (green dots). $^{**}P < 0.01$; $^{***}P < 0.001$ by paired t-test.
